# The effects of vagus nerve stimulation on the course and outcomes of patients with bipolar disorder in a treatment-resistant depressive episode: a 5-year prospective registry

**DOI:** 10.1186/s40345-020-0178-4

**Published:** 2020-05-02

**Authors:** R. Hamish McAllister-Williams, Soraia Sousa, Arun Kumar, Teresa Greco, Mark T. Bunker, Scott T. Aaronson, Charles R. Conway, A. John Rush

**Affiliations:** 1 Northern Centre for Mood Disorders, Newcastle University, Newcastle upon Tyne, UK; 2 Regional Affective Disorders Service, Cumbria, Northumberland Tyne and Wear NHS Foundation Trust, Newcastle upon Tyne, UK; 3 LivaNova USA PLC, Houston, TX USA; 4 Department of Clinical Research, Sheppard Pratt Health System, Baltimore, MD USA; 5 Department of Psychiatry, Washington University School of Medicine in St. Louis, St. Louis, MO USA; 6 Duke-National University of Singapore, Singapore, Singapore; 7 Department of Psychiatry and Behavioral Sciences, Duke University School of Medicine, Durham, NC USA; 8 Department of Psychiatry, Health Sciences Center, Texas Tech University Permian Basin, Midland, TX USA

**Keywords:** Bipolar disorder, Depression, Vagus Nerve Stimulation Therapy, VNS TRD registry, Response, Suicidality, Treatment-resistant depression

## Abstract

**Background:**

To compare illness characteristics, treatment history, response and durability, and suicidality scores over a 5-year period in patients with treatment-resistant bipolar depression participating in a prospective, multicenter, open-label registry and receiving Vagus Nerve Stimulation Therapy (VNS Therapy) plus treatment-as-usual (VNS + TAU) or TAU alone.

**Methods:**

Response was defined as ≥ 50% decrease from baseline Montgomery–Åsberg Depression Rating Scale (MADRS) total score at 3, 6, 9, or 12 months post-baseline. Response was retained while MADRS score remained ≥ 40% lower than baseline. Time-to-events was estimated using Kaplan–Meier (KM) analysis and compared using log-rank test. Suicidality was assessed using the MADRS Item 10 score.

**Results:**

At baseline (entry into registry), the VNS + TAU group (N = 97) had more episodes of depression, psychiatric hospitalizations, lifetime suicide attempts and higher suicidality score, more severe symptoms (based on MADRS and other scales), and higher rate of prior electroconvulsive therapy than TAU group (N = 59). Lifetime use of medications was similar between the groups (a mean of 9) and was consistent with the severe treatment-resistant nature of their depression. Over 5 years, 63% (61/97) in VNS + TAU had an initial response compared with 39% (23/59) in TAU. The time-to-initial response was significantly quicker for VNS + TAU than for TAU (p < 0.03). Among responders in the first year after implant, the KM estimate of the median time-to-relapse from initial response was 15.2 vs 7.6 months for VNS + TAU compared with TAU (difference was not statistically significant). The mean reduction in suicidality score across the study visits was significantly greater in the VNS + TAU than in the TAU group (p < 0.001).

**Conclusions:**

The patients who received VNS + TAU included in this analysis had severe bipolar depression that had proved extremely difficult to treat. The TAU comparator group were similar though had slightly less severe illnesses on some measures and had less history of suicide attempts. Treatment with VNS + TAU was associated with a higher likelihood of attaining a response compared to TAU alone. VNS + TAU was also associated with a significantly greater mean reduction in suicidality.

**Limitations:**

In this registry study, participants were not randomized to the study treatment group, VNS Therapy stimulation parameters were not controlled, and there was a high attrition rate over 5 years.

*Trial registration* ClinicalTrials.gov NCT00320372. Registered 3 May 2006, https://clinicaltrials.gov/ct2/show/NCT00320372 (retrospectively registered)

## Background

Patients with bipolar disorder are symptomatic about 50% of the time, the vast majority of which is depression (Judd et al. 2002, 2003). However, treatment options for bipolar depression are limited. For example, the UK National Institute for Health and Social Care (NICE) guidelines for the management of bipolar depression list just 3 treatments that are supported by replicated randomized controlled trials: lamotrigine, quetiapine, and olanzapine (with or without fluoxetine) (National Institute for Health and Care Excellence 2014). Since publication of the NICE guidelines, additional evidence has emerged from randomized controlled trials supporting the efficacy of lurasidone for the acute treatment of bipolar depression (Loebel et al. 2014a, b). This limited number of treatment options for bipolar depression is further compromised as quetiapine and olanzapine are often poorly tolerated due to weight gain and sedation (Calabrese et al. 2005; Tohen et al. 2003).

The clinical challenge of managing bipolar depression is further illustrated by observations of high rates of antidepressant usage (Kessing et al. 2016; Yoon et al. 2018) despite evidence of questionable efficacy (National Institute for Health and Care Excellence 2014; Sidor and Macqueen 2011). The implication is that many patients suffer from treatment-resistant bipolar depression (TRBD). The prevalence of TRBD is unknown due to a lack of a consensus definition (Hidalgo-Mazzei et al. 2019). However, it is known that about 50% and 30% of depressed bipolar patients remain depressed at 6 and 12 months, respectively, following initiation of antidepressant treatment; and the lack of treatment effects is due to non-response, intolerance, or non-acceptance of treatment (Kupfer et al. 2000). As a result, TRBD is the major contributor to the enormous burden of disease associated with bipolar disorder (Ferrari et al. 2016).

Given the significant unmet need with regards to the management of bipolar depression, it is important that alternative treatment options for patients with TRBD are explored. One potential option is Vagus Nerve Stimulation Therapy (VNS Therapy).

VNS Therapy has primarily been examined in unipolar treatment-resistant depression (TRD). The largest data set supporting its use in TRD is a 5-year VNS TRD registry of nearly 500 participants (representing both unipolar and bipolar TRD) who received adjunctive VNS Therapy plus treatment-as-usual (VNS + TAU). In this registry, the VNS-implanted TRD participants were compared with 300 other TRD participants with similar clinical presentations who received only TAU (Aaronson et al. 2017). It is important to note that the participants included in the registry were not randomized to VNS + TAU or TAU. Rather, treatment was determined by a participant’s choice and availability of VNS Therapy.

The data from the VNS TRD registry revealed that the adjunctive VNS Therapy group had significantly higher 5-year cumulative response (67.6% vs 40.9%) and remission (43.3% vs 25.7%) rates compared to the TRD patients who received TAU alone (Aaronson et al. 2017). Additionally, VNS + TAU led to a more durable response as the time-to-relapse from initial response for responders in the first year was 10.1 months versus 7.3 months for participants receiving TAU alone (Kumar et al. 2019). Safety assessment in the registry also found a greater reduction in suicidality in participants receiving VNS + TAU compared to TAU alone (Aaronson et al. 2017).

Nierenberg and colleagues have previously described the outcomes of 25 patients with TRBD who were included in acute and long-term early studies of VNS Therapy for the treatment of depression (Nierenberg et al. 2008). The authors reported that the antidepressant efficacy outcomes for these TRBD patients were similar to the unipolar TRD patients.

Benefit of VNS Therapy in patients with bipolar disorder is also supported by a published case series that included 5 patients who demonstrated sustained improvement in depressive symptoms and a lack of manic episodes during the follow-up period; and 3 of these patients were followed for about 5 years (Oldani et al. 2015).

In this report—using the 5-year VNS TRD registry discussed above—we examine the pre-treatment clinical characteristics and the clinical outcomes in a subgroup of TRD patients with TRBD comparing VNS + TAU versus TAU alone based on the following areas of interest:

I. Illness characteristics and previous treatments received prior to inclusion in the registry
II. Cumulative depressive symptom response (defined by ≥ 50% reduction in Montgomery–Åsberg Depression Rating Scale [MADRS]) over the 5-year registry observation period
III. Duration of response (defined a priori as maintenance of ≥ 40% reduction from baseline MADRS)
IV. Change in suicidality score over the 5-year registry observation period

## Methods

### Study population

Analysis of the 5-year VNS TRD registry data set described here included 156 participants with bipolar disorder (both bipolar I and II disorders): n = 97 received VNS + TAU and n = 59 received TAU. To be eligible to participate in the VNS TRD Registry, participants had to be over 18 years of age, experiencing an active major depressive episode of 2 years or longer in duration (either unipolar or bipolar), or had a history of at least 3 major depressive episodes, including the current depressive episode, and a history of inadequate response to 4 or more adequate antidepressant treatments (dosage per Physicians’ Desk Reference labeling for a minimum of 4 weeks), which could include electroconvulsive therapy (ECT). Participants could not have a history of a psychotic disorder or rapid-cycling bipolar disorder, or psychotic features in the present major depressive episode. A more detailed list of study entry criteria can be found elsewhere (Aaronson et al. 2017; Olin et al. 2012). ClinicalTrials.gov Identifier: NCT00320372.

### Study treatment

Before enrollment into the VNS TRD Registry, participants could select the treatment group of their choice (ie, TAU or VNS + TAU). The exception to this were those VNS + TAU subjects who entered the registry via rollover from a previous flexible dose-finding VNS trial (Aaronson et al. 2013). Some participants could be assigned to receive the alternate treatment by the site for various reasons, including availability of surgical implantation at a site, number of allocated slots for implantation, or failure to qualify for insurance reimbursement or VNS Therapy implantation. Device implantation surgery and related medical care were covered either by a participant’s insurance policy or from personal funds.

### Assessments

The assessment of the registry participants included in this analysis has been detailed elsewhere (Aaronson et al. 2017). Participants in the VNS + TAU group underwent implantation during Visit 2 (baseline). Post-baseline follow-up visits for all participants were conducted at 3, 6, 9, 12, 18, 24, 30, 36, 42, 48, 54, and 60 months. The primary measure of depression for this registry was the MADRS (Carmody et al. 2006) which was administered by central blinded raters. Other psychiatric outcome measures were the Quick Inventory of Depressive Symptomatology–Self Report (QIDS-SR) (Trivedi et al. 2004; Rush et al. 2003) and the Clinical Global Impression (CGI) scale (Guy 1976).

### Statistical analysis

The intent-to-treat (ITT) population included 195 registry participants with bipolar disorder (n = 134 VNS + TAU; n = 61 TAU) defined as those who completed their baseline visit, received their respective treatment, and completed at least one post-baseline assessment. To ensure consistent VNS Therapy dose and follow-up schedule, the analysis sample excluded individuals who were "crossed over" from VNS Therapy treatment in the previously reported flexible dose study (n = 37) since most of these participants had consistent follow-up data for only 1 year (Aaronson et al. 2013). In addition, we excluded participants who had a baseline MADRS score < 10 indicating that they were already remitted from their major depressive episode (Zimmerman et al. 2004); this excluded n = 2 from the TAU group. The remaining 156 TRBD patients comprised of N = 97 receiving VNS + TAU and N = 59 receiving TAU and were included in the analysis described here. Note that participants who were crossed over to another treatment group during the study were censored at the last visit before cross-over.

Time-to-initial response was defined as the time from baseline to the first visit when there was reduction in MADRS score of ≥ 50% compared to baseline. A probability of time-to-initial response was estimated using Kaplan–Meier (KM) method. KM probability estimates were calculated for the time-to-event with 95% confidence intervals at 3, 6, 9, and 12 months. Time-to-event curves for the 2 treatment groups were compared using Log-rank test. A Cox proportional-hazard model was used to estimate the hazard ratio (and 95% confidence interval) of the instantaneous chance of a participant having an event in the VNS + TAU group compared to the TAU group at any given time during follow-up.

Given the different proportion of participants with bipolar I or II disorder between the VNS + TAU and TAU groups, a second Cox proportional-hazard model was used to evaluate the time-to-first response, adjusting for the effects of bipolar diagnosis and interaction between treatment and bipolar diagnosis.

Persistence of response was defined as an ongoing reduction in MADRS score of ≥ 40% after an antidepressant response was recorded (reduction of baseline MADRS of ≥ 50%). Persistence of response was calculated for all study participants who had an initial response in the first year of study treatment. Participants were categorized in subgroups by the visit when the initial response occurred. A KM analysis was performed to compare the retention of response in VNS + TAU and TAU alone in a time-to-event analysis framework.

Participants were considered severely suicidal if they had a score of ≥ 4 on MADRS Item 10. The percentage who were still severely suicidal was calculated for each post-baseline visit. Similarly, the percentage who were non-severely suicidal at baseline who became severely suicidal was calculated for each post-baseline visit. Average change in suicidality score for VNS + TAU and TAU on MADRS Item 10 is presented for each post-baseline visit.

If there were 1 or 2 consecutive missing data, then the data was imputed with the average of the 2 adjacent non-missing data. No imputation was done for 3 or more consecutive missing data points. After imputation, participants were censored at the last visit with non-missing data for all the analysis. Thus, there were a total of 412 visits with data for TAU group and 856 visits with data for VNS + TAU group in the censored data set. Imputation for a single missed data point in the censored data set was done for 32 visits (32/412 [7.8%]) of the available data for TAU group and for 59 visits (59/856 [6.9%]) of the available data for VNS + TAU group. Imputation for 2 consecutive missing data points was done for 14 visits (14/412 [3.4%]) of the available data for TAU group and for 28 visits (28/856 [3.3%]) of the available data for VNS + TAU group. Overall, there were 46 imputed data (46/412 [11.2%]) of available data in TAU group and 87 imputed data (87/856 [10.2%]) of all available data) in VNS + TAU group.

This imputation method has desirable properties as detailed in Kumar et al. (2019). The data set has a regular response pattern (when defined as reduction of MADRS score of ≥ 50%), ie, the same response at the adjacent visits around one missing data: 78.1% for TAU and 62.7% for VNS + TAU, and around 2 consecutive missing data items: 100% for TAU and 78.1% for VNS + TAU. Thus, occurrence of initial or second response could have been altered due to imputation only for 1.7% of the censored data for the TAU group and 3.3% of censored data in the VNS + TAU group. Similarly, the censored data set provided a regular response pattern (when defined as reduction of MADRS score of ≥ 40%) around 1 missing data: 71.9% for TAU and 57.6% for VNS + TAU and around 2 consecutive missing data: 100% for TAU and 78.6% for VNS + TAU. Thus, prolongation of the response maintenance could have occurred in only 2.2% of the censored data in the TAU group and 3.6% of the censored data in the VNS + TAU group. Given this small percentage of data that could have an altered response pattern due to imputation, it was concluded that the imputation method would work well for this data set and that it could not have altered the result substantially in favor of any treatment group.

## Results

### Sample demographics and illness characteristics

Table 1 summarizes demographic information and baseline clinical characteristics for the analysis sample.

**Table 1:** Demographic and baseline clinical characteristics

	VNS + TAU (N = 97)	TAU (N = 59)	*P* *
Mean age ± SD (years)	47.0 ± 10.2	47.8 ± 10.6	0.65
Female, n (%)	72 (74.2%)	47 (79.7%)	0.56
White, n (%)	93 (95.9%)	56 (94.9%)	1
Mean age ± SD at initial onset of depressive symptoms (years)	20.0 ± 11.5	18.9 ± 9.2	0.51
Mean age ± SD at initial diagnosis of depression (years)	26.9 ± 10.6	27.9 ± 11.6	0.59
Lifetime number of diagnosed depressive episode	20.7 ± 29.2	13.7 ± 23.2	0.10
Psychiatric hospitalizations within the 5 years prior to registry enrollment	3.6 ± 5.4	1.5 ± 2.1	< 0.001
Lifetime suicide attempts	2.7 ± 4.8	1.5 ± 2.9	0.05
DSM-IV-TR primary diagnosis, n (%)			
Bipolar I disorder, currently moderately severe major depressive episode	19 (19.6%)	18 (30.6%)	0.17
Bipolar I disorder, currently severe major depressive episode	46 (47.4%)	10 (16.9%)	< 0.001
Bipolar II disorder, currently depressed	32 (33.0%)	31 (52.5%)	0.025
Baseline scores, n (%)			
Montgomery–Åsberg Depression Rating Scale	33.7 ± 7.3	29.7 ± 5.9	< 0.001
Clinical Global Impression—Severity	5.2 ± 0.8	4.7 ± 0.7	< 0.001
Quick Inventory of Depressive Symptomatology-Self Report	18.4 ± 4.9	15.9 ± 5.2	0.004
Suicidality-based on MADRS Item 10	2.7 ± 1.4	2.0 ± 1.2	0.003

*SD* standard deviation

* P-values are from two-sided t-test for comparing means assuming unequal variance or z-test for comparing proportions

The age at onset of depressive symptoms (around 19–20 years of age) and age at initial diagnosis of an episode of depression (around 8 years later) were similar between the groups. Overall, there were significantly higher proportion of participants with a bipolar I diagnosis in the VNS + TAU group (n = 65 [67.0%] vs n = 28 [47.5%]) and lower rate of those with a bipolar II diagnosis (32 [33.0%] vs 31 [52.5%]) compared with the TAU group (Chi-squared test for homogeneity, p = 0.0158). The VNS + TAU group had experienced more episodes of lifetime depressive episodes than the TAU group, though this was not statistically significant. Moreover, the VNS + TAU group had a history of more psychiatric hospitalizations within the 5 years prior to entering the registry and had more lifetime suicide attempts. Further, the VNS + TAU subjects had greater depressive symptomology as assessed by the MADRS, QIDS-SR, and CGI. Additionally, the VNS + TAU group scored significantly higher on the suicidality item of the MADRS Item 10.

Treatment histories are presented in Table 2. There was a very similar distribution of lifetime use of medications. The mean number of lifetime antidepressant treatment courses was approximately 9, with a maximum of 14 in both treatment groups. All study participants had received antidepressants in the past or present, and selective serotonin reuptake inhibitors (SSRIs) and serotonin and norepinephrine reuptake inhibitors (SNRIs) were the most frequently prescribed antidepressant medication classes. With regard to medications specifically recommended in guidelines for bipolar depression (National Institute for Health and Care Excellence 2014), lamotrigine was the drug most commonly prescribed, followed by quetiapine. About half of the VNS + TAU group had taken lithium or sodium valproate, slightly more than seen in the TAU group. Just over half of the VNS + TAU group had prior ECT treatment, with a smaller number in the TAU group (54% vs 39%). Most participants had received psychological therapies, with a lifetime frequency of individual therapy being above 80% in both groups.

**Table 2:** Lifetime treatment histories

	VNS + TAU (N = 97)	TAU (N = 59)
Number of treatment courses*		
Mean	9.2	9.0
Maximum	14	14
Minimum	3	4
Antidepressants, n (%)	97 (100%)	59 (100%)
Bupropion	71 (73%)	38 (64%)
Selective serotonin reuptake inhibitors (SSRIs)	88 (91%)	50 (85%)
Serotonin and norepinephrine reuptake inhibitors (SNRIs)	78 (80%)	48 (81%)
Other	68 (70%)	35 (59%)
Antipsychotics, anticonvulsants, and other medications, n (%)		
Lamotrigine	62 (64%)	44 (75%)
Quetiapine	56 (58%)	35 (59%)
Olanzapine	37 (38%)	24 (24%)
Olanzapine + fluoxetine	5 (5%)	6 (10%)
Lithium	53 (55%)	25 (42%)
Sodium valproate	54 (56%)	20 (34%)
Electroconvulsive therapy, n (%)	53 (54%)	23 (39%)
Psychological therapies, n (%)		
Cognitive behavioral therapy (CBT)	44 (45%)	23 (39%)
Individual therapy	83 (86%)	48 (81%)

* A course of treatment was defined as at least a 4-week continuous period in which a patient used one or more treatments for their depression. A new course of treatment started each time a drug was added or dropped. Courses of treatment were classified as electroconvulsive therapy, monotherapy, combination therapies, augmentation therapies, or other psychiatric treatments

### Cumulative response rates

**Fig. 1:**
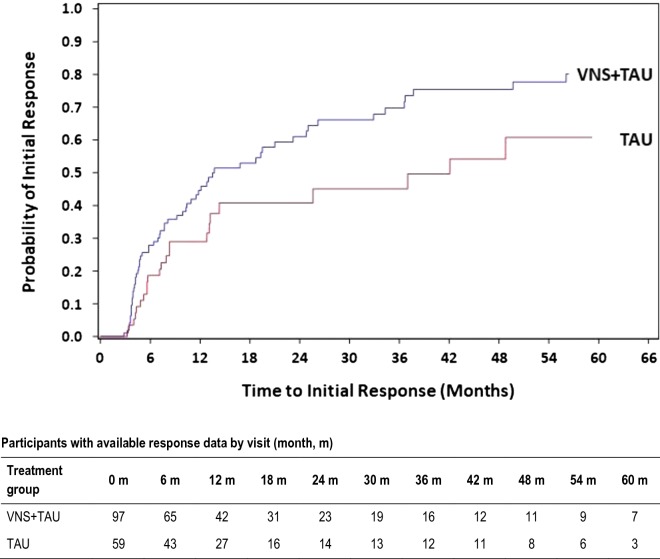
Kaplan–Meier plot for time-to-initial response based on MADRS score

Over the 5-year observation period, 61 of 97 (63%) in the VNS + TAU group had an initial response (defined as ≥ 50% reduction in MADRS from baseline) compared to 23 of 59 (39%) of participants in the TAU group. The KM plot in Fig. 1 shows that time-to-initial response was significantly shorter for VNS + TAU than for TAU alone (p = 0.03 for log-rank test). The estimated cumulative probability for the time-to-initial response was higher for the VNS + TAU group as compared to the TAU group over most of the follow-up period. Median time-to-initial response was 13.7 month (Q1 = 5, Q3 = 37.7) for VNS + TAU group compared to 42.1 months (Q1 = 8.3, Q3 = not estimable) for TAU group. Hazard ratio for time-to-initial response for VNS + TAU compared to TAU was 1.7 (95% CI 1, 2.7) meaning a larger chance for a participant in the VNS + TAU group to get an initial response compared to a participant in the TAU group at any given time during the follow-up, though the hazard ratio was not statistically significant.

The Cox proportional-hazard model on time-to-first response adjusting for the effects of bipolar diagnosis and the correspondent interaction, confirmed the benefit of VNS + TAU in reducing the time-to-first response (HR = 1.6; 95% CI 0.98, 2.7) and VNS + TAU showed trends of effectiveness in both sub-populations (HR = 2.1 in bipolar I and HR = 1.3 in bipolar II, even if a significant treatment effect of VNS + TAU vs TAU was seen just in the participants with bipolar I (95% CI 1.0, 4.3) (Table 3). This may in part be driven by the smaller number of patients with bipolar II vs bipolar I disorder (n = 59 vs n = 97) and the low rate of responses in the bipolar II subgroup (n = 17 vs n = 14 for the VNS + TAU and TAU groups, respectively). Due to the low rate, it was also not possible to estimate the 95% confidence intervals in KM analysis for the median time-to-first response in the bipolar II patients (Table 4).

**Table 3:** Cox proportional-hazards model examining effect of bipolar diagnosis on the time-to-first response

Effect	Hazard ratio	95% CI lower limit	95% CI upper limit	p-value
VNS + TAU vs TAU	1.6	0.98	2.7	0.06
Bipolar II vs bipolar I	0.96	0.6	1.6	0.9
VNS vs TAU in bipolar I	2.1	1.0	4.3	0.04
VNS vs TAU in bipolar II	1.3	0.6	2.6	0.5

**Table 4:** Kaplan–Meier estimates for time-to-first response, months

	First quartile (95% CI)	Median (95% CI)	Third quartile (95% CI)
Bipolar 1			
VNS + TAU	5.8 (4.1, 7.7)	13 (7.7, 23.2)	36.6 (23.2, NE)
TAU	13.1 (4, 37)	37 (13.1, NE)	NE (37, NE)
Bipolar 2			
VNS + TAU	4.7 (3.7, 10.4)	19.5 (9.2, NE)	NE (24.8, NE)
TAU	7.9 (4.3, 13.2)	14.3 (8.3, NE)	NE (48.8, NE)

*CI* confidence interval, *NE* not estimable

### Duration of response

Maintenance of response was defined a priori as maintenance of ≥ 40% reduction from baseline MADRS and assessed in those who showed a response in the first year of follow-up. In the VNS + TAU group, 46 of the 61 responders (75.4%) responded in the first year; and in the TAU group, 19 of the 23 responders (82.6%) responded in the first year. Numbers are small and hence comparisons between the 2 groups may not be robust.

A KM analysis of the data estimated that the median time-to-relapse from initial response in the first year was 15.2 months (Q1 = 6.7, Q3 = 25.4) for the VNS + TAU group compared with 7.6 months (Q1 = 3.4, Q3 = 14.7) for the TAU group. The hazard ratio for relapse after the initial response was 0.7 (95% CI 0.3, 1.4) in favor of VNS, though this was not statistically significant. In terms of actual data, it was possible to examine maintenance of response 6 months after initial response in participants who demonstrated an initial response at the 3-, 6-, or 12-month study visits. Of these, 30/39 (76.9%) in the VNS + TAU group were maintaining a response 6 months later, compared with 10/18 (55.6%) in the TAU group. There was limited data to examine maintenance of response 12 months after initial response since this was only available for those who showed an initial response at the 6- or 12-month visits. However, again, the proportion maintaining a response was numerically higher in the VNS + TAU compared with TAU group (6/13 [46.1%] vs 3/11 [27.3%], respectively).

### Suicidality

**Fig. 2:**
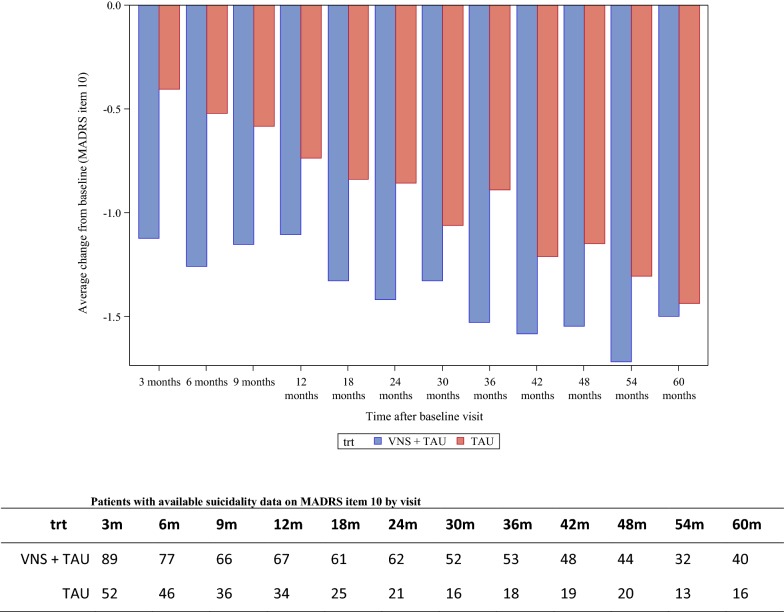
Mean change in suicidality score from baseline based on MADRS Item 10

A total of 33 (33/97; 34%) in the VNS + TAU group and 8 (8/59; 14%) in the TAU group were severely suicidal at baseline based on MADRS (a score ≥ 4 on MADRS Item 10 corresponding to the responses “probably better off dead” and “active preparations for suicide”). Notably, the mean reduction in suicidality score across the study visits was significantly greater in the VNS + TAU than in the TAU group (P < 0.001 as per F-test) (Fig. 2).

In each treatment group, the percentage who became severely suicidal post-baseline was less than 15% (Table 5) and the difference between the treatment groups was not statistically significant.

**Table 5:** Change in suicidal rating from non-severe to severe based on MADRS Item 10

Visit months	VNS + TAU	TAU
3	3/57 (5.3%)	3/44 (6.8%)
6	2/49 (4.1%)	4/40 (10%)
9	1/43 (2.3%)	2/32 (6.3%)
12	6/44 (13.6%)	2/28 (7.1%)
18	1/39 (2.6%)	1/22 (4.5%)
24	0/36 (0%)	0/19 (0%)
30	1/29 (3.4%)	0/14 (0%)
36	1/30 (3.3%)	0/16 (0%)
42	1/28 (3.6%)	1/15 (6.7%)
48	2/27 (7.4%)	1/18 (5.6%)
54	1/18 (5.6%)	1/10 (10%)
60	2/23 (8.7%)	0/14 (0%)

The numerator denotes the number of participants who had a non-severe suicidal rating at baseline (score < 4) and developed a severe suicidal rating (score ≥ 4) at a post-baseline visit based on MADRS Item 10. The denominator denotes the number of participants who had a non-severe suicidal rating at baseline and attended a post-baseline visit

## Discussion

Given the frequency of TRBD and its impact on patients with bipolar disorder, it is important to consider all possible treatment options. This post-hoc analysis suggests that in a non-randomized study following the outcomes of patients with TRBD for up to 5 years, the addition of VNS Therapy to TAU had significantly greater cumulative response rates, faster onset of antidepressant response, and the responses were longer in duration than in participants receiving TAU alone. Critically, VNS + TAU was also associated with a significantly greater reduction in suicidal ideation compared with TAU alone, despite the VNS + TAU group being more severely depressed at baseline and with high ratings of suicidality. These findings are consistent with the observations made in a much larger group of patients with unipolar or bipolar depression (Aaronson et al. 2017). They are also consistent with a previous post-hoc analysis of 25 patients with TRBD who made up 11% of a larger TRD population who received VNS Therapy alongside TAU in a sham-controlled acute study with long-term open-label follow-up (Nierenberg et al. 2008). The only other study of the safety and efficacy of VNS Therapy in bipolar disorder is a 1-year pilot study of VNS Therapy in 9 patients with rapid cycling bipolar disorder that did not have a comparator group (Marangell et al. 2008).

These findings suggest that VNS Therapy may be effective in patients with very significant difficult to treat depression in the context of bipolar disorder. Those treated with VNS Therapy had an average of 20.7 lifetime episodes of depression, 3.6 psychiatric hospitalizations in the previous 5 years, and 2.7 lifetime suicide attempts. They had received an average of 9 mediation treatment courses over their lifetime and all had received an antidepressant, despite the lack of evidence that these are efficacious in patients with bipolar disorder (Sidor and Macqueen 2011; Young et al. 2010). The vast majority had also received psychotherapy, and about half (54%) had been treated with ECT. Importantly, despite the VNS + TAU participants having considerably more severe depressive histories (statistically significantly more severe depressive symptomology and greater suicidal ideation at baseline prior to treatment), the VNS + TAU group demonstrated superior antidepressant outcomes.

The magnitude of the effect on cumulative response rates with VNS + TAU versus TAU was slightly larger than that seen in patients with unipolar depression in the original analysis of this data set (Aaronson et al. 2017). However, the assessment of the impact of VNS Therapy on durability of response in this current analysis is not as great as that seen in the unipolar patients studied as part of this registry (Kumar et al. 2019). This is perhaps not surprising given that bipolar disorder is more recurrent than unipolar disorder (Angst et al. 2003). While there was no significant difference in durability of response between the VNS + TAU and TAU groups in this analysis, numerically the participants receiving VNS + TAU did better. The lack of significant findings with regards to durability of response may have in part arisen due to the small numbers of patients included in the analysis, particularly at later visit time points, and the relative infrequency of assessment of mood symptoms. Given the importance of prophylaxis in a recurrent disorder such as bipolar disorder, further research investigating the prophylactic efficacy of VNS Therapy is indicated, including in patients with rapid cycling, utilizing frequent assessments of symptoms.

Previous analyses in a mixed, but predominantly unipolar TRD population, have suggested a reduction in rates of suicide and all-cause mortality associated with VNS treatment (Aaronson et al. 2017; Feldman et al. 2013). The significant reductions in suicidality seen in this post-hoc analysis of patients with TRBD treated with VNS Therapy suggests that such findings might be expected in a larger population of individuals with TRBD, though further research is required. Similarly, it is important to further explore whether the cost effectiveness of VNS Therapy, observed in mixed TRD populations (predominantly unipolar depression), will also be observed in TRBD patients (Feldman et al. 2013).

VNS Therapy is generally well tolerated as revealed in a meta-analysis of over 1000 patients with either unipolar or bipolar depression (Berry et al. 2013). Data regarding the impact of adverse effects of medication was not available in the specific sub-sample of bipolar patients reported here. However, in the complete sample of unipolar and bipolar patients in the registry study (Aaronson et al. 2017), medication adverse effects were assessed using the frequency, intensity, and burden of side effects rating (FIBSER) scale (Wisniewski et al. 2006). Based on this scale, the patients in the VNS + TAU group reported higher scores for frequency, severity, and burden of side effects at baseline, but at the 12 and 24 months timepoints, there were no significant differences between the groups (data available on request).

There was a significant difference in the proportion of bipolar I participants in the two groups (67% in TAU + VNS vs 47% in TAU) and it is possible that this, in part, impacted the results. A significant effect of VNS + TAU over TAU was seen for time-to-first response in bipolar I participants (HR = 2.1; 95% CI 1, 4.3). This was not evident in those with bipolar II disorder, though the event rate was such that it is not possible to draw meaningful conclusions regarding a bipolar I vs bipolar II difference in the effectiveness of VNS Therapy added to TAU. In addition, this registry study unfortunately did not collect formal ratings of manic symptoms, so it is not possible to infer the effects of VNS Therapy on elevated mood. A previous 12-month follow-up study of VNS Therapy that included 20 patients with bipolar disorder assessed manic symptoms (Rush et al. 2005). Two of the participants developed brief mild manic episodes that lasted 1 to 2 weeks, and there were two short periods of sub-syndromal hypomanic symptoms (about 1 to 3 days), during the first 3 months of treatment with VNS Therapy. One participant (with a baseline diagnosis of unipolar disorder) developed a manic episode during the subsequent 9 months of treatment with VNS Therapy. Additional data are required to address whether there are potential differential effects between bipolar I vs II and the effect of VNS Therapy on hypomanic/manic symptoms, and such data will hopefully become available following completion of the current ongoing RECOVER randomized trial in the USA and the RESTORE-LIFE registry in Europe.

The study had several additional limitations. Participants were not randomized to the treatment groups, and when VNS Therapy was an available treatment option, there appeared to be a tendency for the treatment to be utilized in patients with bipolar disorder who had a significant degree of pharmacological non-response (or intolerance) and who had a higher rate of ECT treatment history (54%). This rate of ECT usage is similar to that seen in the unipolar patients included in the registry (61%) who received VNS. In addition, there was no sham VNS for the “TAU” group. Therefore, it is not possible to conclude with high certainty that all the effects observed are exclusively related to treatment with adjunctive VNS Therapy. The higher baseline MADRS score in the VNS + TAU compared with TAU group might also mean that regression to the mean may have played a larger role in the VNS + TAU group. In this effectiveness trial, medications and all other treatments, such as TMS and ECT, could change during treatment for either treatment group. Furthermore, study participants and clinicians were knowledgeable about the care being given. However, the off-site central raters collecting the MADRS data were blind to both treatment group and the overall clinical status of the study participants. The population examined limits generalizability, though it is of course reasonably representative of participants suffering from a significant degree of difficult to treat depression in the context of bipolar disorder. Suicidality was not assessed using a specific suicidality scale, but rather a single item in the MADRS. Finally, in this 5-year longitudinal study, the participant attrition over time limits our ability to address with significant sample sizes some of the questions that are posed.

## Conclusions

VNS Therapy as an adjunctive treatment to TAU was more effective than TAU alone in reducing depressive symptomatology, and led to a greater reduction in suicidal ideation, and, on average, a more rapid antidepressant response. Further, the antidepressant effects observed in the VNS + TAU group vis-à-vis TAU were likely more durable. Together, these findings support previously observed findings that adjunctive VNS is an efficacious antidepressant treatment in very severe, treatment-resistant bipolar depression.

## Abbreviations

CGI: Clinical Global Impression
ECT: Electroconvulsive therapy
ITT: Intent-to-treat
KM: Kaplan–Meier
MADRS: Montgomery–Åsberg Depression Rating Scale
QIDS-SR: Quick Inventory of Depressive Symptomatology–Self Report
TAU: Treatment-as-usual
TRBD: Treatment-resistant bipolar depression
TRD: Treatment-resistant depression
VNS Therapy: Vagus Nerve Stimulation Therapy

## Availability of data and materials

The datasets and results related to the VNS TRD registry that are open access can be found at https://clinicaltrials.gov/ct2/show/NCT00320372. De-identified participant’s data for the VNS TRD Registry will not be shared. Please contact the corresponding author for data requests.
